# Iron metabolism and ferroptosis in nonalcoholic fatty liver disease: what is our next step?

**DOI:** 10.1152/ajpendo.00260.2023

**Published:** 2024-03-20

**Authors:** Xiang Shen, Ziqi Yu, Changli Wei, Chong Hu, Jianyong Chen

**Affiliations:** ^1^Munich Medical Research School, Ludwig Maximilian University of Munich, Munich, Germany; ^2^Department of Gastroenterology and Hepatology, The First Affiliated Hospital of Nanchang Medical College, Jiangxi Provincial People's Hospital, Nanchang, People’s Republic of China

**Keywords:** ferroptosis, iron metabolism, lipid peroxidation, NAFLD, NASH

## Abstract

Nonalcoholic fatty liver disease (NAFLD) is the most common chronic liver disease with increasing prevalence worldwide. NAFLD could develop from simple hepatic steatosis to nonalcoholic steatohepatitis (NASH), NASH-related fibrosis, cirrhosis, and even hepatocellular carcinoma. However, the mechanism of NAFLD development has not yet been fully defined. Recently, emerging evidence shows that the dysregulated iron metabolism marked by elevated serum ferritin, and ferroptosis are involved in the NAFLD. Understanding iron metabolism and ferroptosis can shed light on the mechanisms of NAFLD development. Here, we summarized studies on iron metabolism and the ferroptosis process involved in NAFLD development to highlight potential medications and therapies for treating NAFLD.

## INTRODUCTION

Nonalcoholic fatty liver disease (NAFLD) is the most common chronic liver disease constituting a significant burden on global healthcare. It is estimated to affect ∼25% of the world’s population (1–3). It is clinically heterogeneous with a broad spectrum of conditions ranging from simple hepatic steatosis to nonalcoholic steatohepatitis (NASH) (4–6). NASH is a severe form of NAFLD characterized by hepatic lipid accumulation, oxidative stress, cell death, inflammatory infiltration, and the potential progression to fibrosis, cirrhosis, end-stage liver disease, or even hepatocellular carcinoma (5, 7, 8). Although, the prognosis of simple hepatic steatosis could be benign (7), without intervention, nearly 25% of patients with NAFLD will develop NASH without fibrosis (9). Furthermore, 20–30% of the cases progress to NASH with fibrosis within 3 years (9, 10). For patients already developed NASH, more than 33% of the patients will develop NASH-related cirrhosis (9).

The mechanisms underlying the progression from NAFLD to NASH are still not fully elucidated. Various risk factors contribute to NAFLD, including insulin resistance, obesity, metabolic syndromes, oxidative stress, inflammation, etc. (11). Recent evidence suggests that iron overload plays a role in the process and progression of NAFLD (11, 12). The ferroptosis caused by iron overload was also thought to be one of the hallmarks of the NAFLD (12).

Ferroptosis is a novel iron-dependent programmed cell death characterized by iron-driven lipid peroxidation (13). Morphologically, ferroptosis is characterized by rounded shape, shrinkage of mitochondria with increased membrane density, rupture of the outer mitochondrial layer, reduced cristae but no chromatin condensation (13–15), which is distinguishable from other cell death such as necroptosis, apoptosis, or autophagy.

## IRON METABOLISM AND HOMEOSTASIS

### Iron Absorption

**Figure 1.**
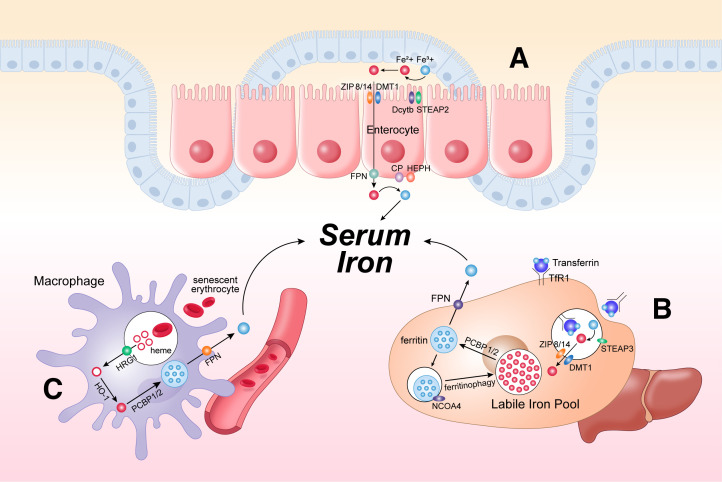
Iron homeostasis and metabolism. *A*: dietary iron absorption in enterocytes. *B*: cellular iron storage. *C*: iron recycling in macrophages by phagocytosis of senescent erythrocytes. CP, ceruloplasmin; Dcytb, duodenal cytochrome b; DMT1, divalent metal transporter 1; FPN, ferroportin; HEPH, hephaestin; HO-1, heme oxygenase 1; HRG1, heme transporter; NCOA4, nuclear receptor coactivator 4; PCBP, chaperones poly (rC) binding protein; STEAP2, six-transmembrane epithelial antigen of the prostate 2; TfR1, transferrin receptor 1; ZIP 8/14, zinc iron regulatory protein family 8/14.

Dietary iron absorption accounts for only 10% of total iron consumption, making it a minor source. The absorption of iron occurs in the duodenum. The duodenal cytochrome b (Dcytb) and six-transmembrane epithelial antigen of the prostate 2 (STEAP2) reduce the ferric iron (Fe^3+^) into ferrous iron (Fe^2+^), which is then transferred by divalent metal transporter 1 (DMT1) or zinc iron regulatory protein family 8/14 (ZIP 8/14) into the enterocytes. The ferroxidase hephaestin (HEPH) and ceruloplasmin (CP) then oxidize ferrous iron back into ferric iron. Subsequently, it is exported to the systemic circulation by the iron exporter ferroportin (FPN) (16, 17) (Fig. 1*A*).

### Iron Storage

The ferric iron in the circulation is then bound to the transferrin. The transferrin-bound iron is then recognized by transferrin receptor 1 (TfR1) to initiate the endocytosis. Within the acidic lysosome, ferric iron is reduced to ferrous iron by six-transmembrane epithelial antigens of the prostate 3 (STEAP3) (18). DMT1 and ZIP 8/14 then transfer the ferrous into cytoplasm to build up the labile iron pool (LIP). These labile irons can then be stored into ferritin with the assistance of chaperones poly (rC) binding protein (PCBP) 1 and 2 (19). The stored ferric iron within ferritin can be either released to LIP by the cargo protein nuclear receptor coactivator 4 (NCOA4) through ferritinophagy, or exported by FPN (19) (Fig. 1*B*).

### Iron Recycling

The iron recycling through macrophages is the major source of iron consumption in human. The macrophages can recognize and capture senescent erythrocyte and undergo phagocytosis. This kind of phagocytosis is also called erythrophagocytosis. Within the phagolysosomes, hemoglobin and heme can be degraded. The heme transporter HRG1 localized on the phagolysosomal membrane then transports the heme across the phagosome membrane to the cytoplasm (20). Heme is then cleaved by heme oxygenase 1 (HO-1) into biliverdin, carbon monoxide, and ferrous iron (21). The ferrous iron is then stored into the ferritin by PCBP1/2 again, exported by FPN, and reduced by HEPH/CP into ferric iron in the circulation (22) (Fig. 1*C*).

### Regulation of Iron Homeostasis

Hepcidin, primarily produced in the liver, serves as the key regulator of iron homeostasis. To become the 25-amino-acid bioactive form, it must undergo proteolytic cleavage (23). It can reduce the iron efflux in circulation by regulating the FPN on the cell surface. After binding the FPN, it can induce the internalization, ubiquitination, and degradation of FPN and decline the export of iron to the circulation. It is also reported that hepcidin may downregulate DMT1 transcription to reduce the ferrous iron uptake at duodenum (24).

## FERROPTOSIS

Dysfunction in iron metabolism may result in iron overload, triggering lipid peroxidation and inducing ferroptosis.

Polyunsaturated fatty acids (PUFAs) such as arachidonic acid (AA) or adrenal acid (AdA) play a key role in the phospholipids involved in ferroptosis. These PUFAs are first catalyzed by acyl-CoA synthetase long-chain family member 4 (ACSL4) to form PUFA-CoAs, which are then esterified by Lysophosphatidylcholine Acyltransferase 3 (LPCAT3) into phosphatidylethanolamines (PUFA-PEs). These PUFA-PEs are then synthesized into phospholipid hydroperoxides (PUFA-PLOOHs) by nonenzymatic reactions driven by Fenton reactions. Lipoxygenases (LOXs) are nonheme iron-dependent dioxygenases. LOX5, LOX12, and LOX15 directly participate in the ferroptosis and catalyze the generation of phospholipid hydroperoxide in the enzymatic way. The phospholipid hydroperoxide is the key inducer of ferroptosis (12, 25–27).

**Figure 2.**
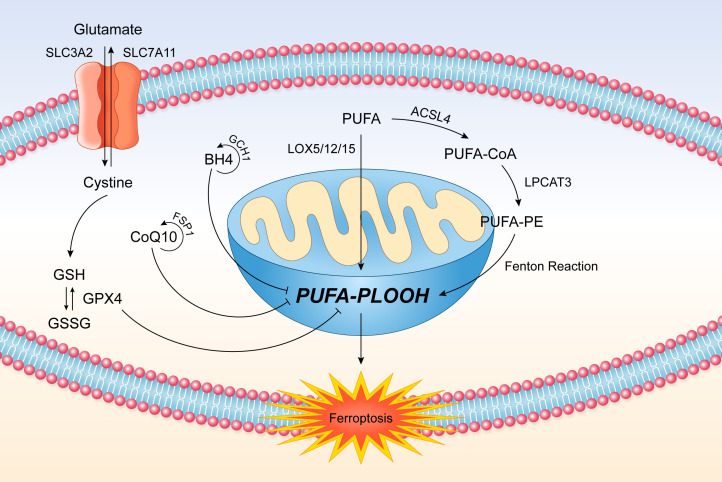
Ferroptosis process. The ferroptosis is caused by the lipid peroxidation. GPX4, CoQ10 and BH4 axis are the main three regulatory axis of the process. ACSL4, acyl-CoA synthetase long-chain family member 4; BH4, 5,6,7,8-tetrahydrobiopterin; FSP1, ferroptosis suppressor protein 1; GCH1, GTP cyclohydrolase I; GPX, glutathione peroxidase; LOX, lipoxygenase; PE, phosphatidylethanolamine; PLOOH, phospholipid hydroperoxide; PUFA, polyunsaturated fatty acid; SLC3A2, solute carrier family 3 membrane 2; SLC7A11, solute carrier family 7 member 11.

Ferroptosis is mainly regulated by the SystemXc^−^/GSH/glutathione peroxidase (GPX)4 axis, ferroptosis suppressor protein 1 (FSP1)-Coenzyme Q10(CoQ10)-NAD(P)H axis, and GTP cyclohydrolase I (GCH1)/5,6,7,8-tetrahydrobiopterin (BH4) axis (Fig. 2).

### SystemXc^−^/GSH/GPX4 Axis

SystemXc^−^ comprises of a regulatory subunit solute carrier family 3 membrane 2 (SLC3A2) and a catalytic solute carrier family 7 member 11 (SLC7A11), which facilitates the uptake of cystine by export of glutamate. The uptaken cystine is then catalyzed by glutamate-cysteine ligase and glutathione synthase into GSH, the substrate of GPX4. GPX4 is the selenium protein that can scavenge lipid peroxide (PUFA-PLOOH) into lipid alcohol (PUFA-PLOH) to prevent ferroptosis (5, 25).

### FSP1-CoQ10-NAD(P)H Axis

As a lipophilic electron carrier, CoQ10 is an antioxidant agent that can serve as a lipophilic radical trapping antioxidant (RTA) guard (28). Ferroptosis suppressor protein 1 (FSP1) can reduce and regenerate CoQ10 by NAD(P)H. This redox cycle of CoQ10 can then suppress the lipid peroxidation and suppress the ferroptosis.

### GCH1/BH4 Axis

5,6,7,8-Tetrahydrobiopterin (BH4) is crucial cofactor in a couple of key metabolic enzymes such as tryptophan hydroxylases, nitric oxide synthases, alkylglycerol monooxygenases, etc. (27). Insufficient BH4 would cause production of oxidative radicals (29). GTP cyclohydrolase I (GCH1) is involved in the production of BH4 and helps the synthesis of BH4 to reduce the oxidative radicals.

### Other Regulators

In addition to the aforementioned axes, the tumor suppressor p53 can also regulate ferroptosis. It is reported that p53 plays a bidirectional role in the regulation of ferroptosis, it can repress ferroptosis when the lipid peroxidation damage is mild, whereas induce ferroptosis when the damage is too severe (30). It can induce the expression of FDXR and further catalyze the transfer of electrons from NADPH to cytochrome p450 (31). Furthermore, it can also upregulate SLC25A28 to induce mitochondrial iron overload (32). Besides, it is also reported that p53 can also induce ferroptosis by p53-STA1-Arachidonate 15-Lipoxygenase (ALOX15) axis (33). The way that P53 regulates GPX4 expression is in a different manner. On one hand, it could inhibit GPX4 by inducing GLS2 to catalyze the conversion of glutamine to glutamate (34) or by inhibiting SLC7A11 and diminishing the System Xc^−^/GSH/GPX4 axis (35). On the other hand, it can also increase GPX4 by promoting the p21/GSH pathway to inhibit ferroptosis (36). Moreover, when inhibiting SLC7A11, the expression of ALOX12 can also be upregulated to induce the lipid peroxidation (37). In addition to ALOX12, the lipid peroxidation can be also induced by dipeptidyl peptidase 4 (DPP4)/NADPH Oxidase 1 (NOX1) pathway (38). Moreover, the p53-iPLA2β axis could play an antiferroptosis pathway by clearance of free radicals (39, 40). Its interaction with some noncoding RNA can also regulate ferroptosis.

MAPK/5' AMP-activated protein kinase (AMPK) signaling can also regulate ferroptosis in different ways. Activation of MAPK signaling pathway can increase the accumulation of intracellular iron by augmenting the expression of TfR1 (41) or by degradation of FTH1 (42). Meanwhile, MAPK signaling activation can also reduce the intracellular iron concentration by upregulating the ferroportin (Fpn1) (43, 44). Moreover, MAPK signaling can also regulate the lipid peroxidation to control ferroptosis. It can induce the lipid peroxidation by upregulating ACSL4 (45) or ALOX15 (46), whereas it can also inhibit lipid peroxidation by inducing the expression of SLC7A11 (47). When MAPK is inhibited, the liver kinase B1 (LKB1)-AMPK signaling will then be activated. Activated AMPK signaling was reported to increase ferroptosis by inducing phosphorylation of Beclin-1 (BECN1) (48), which then in turn bind and inhibit SLC7A11 from System Xc^−^ axis. Furthermore, it is also reported to induce ferroptosis by inducing autophagy (49, 50) or inhibit the mTOR pathway (51, 52).

Nuclear factor erythroid 2-related factor 2 (Nrf2) signaling can also mediate the antioxidant defense. Upon activation, it can activate the downstream antioxidative response element (ARE)-containing gene expression. These genes may promote GSH synthesis, depress the peroxidative production, thus serve as a negative regulator of ferroptosis.

Moreover, the epigenetic regulators such as H3K27me3 (53) and H3K9me3 (54) were also reported to control ferroptosis by affecting transcription, posttranscription, or posttranscriptional modification of ferroptosis-related genes.

## IRON METABOLISM AND FERROPTOSIS IN NAFLD

The dysregulated iron homeostasis was found by different independent groups. Back in the year 2004, Bugianes et al. (55) reported increased ferritin level in patients with severe histological damage. In the year 2010, Valenti et al. (56) also observed iron accumulation in hepatocytes in patients with NAFLD with severe liver damage. In the same year, Nelson et al. (57) reported distinct histological features of hepatic iron deposition in patients with NAFLD. They found the patients with stainable hepatic iron in the reticuloendothelial system cell pattern are associated with advanced fibrosis (57). Research in 2012 in a large cohort of 628 individuals exhibited the association between high serum ferritin level (more than 1.5 times the upper limit of normal) and severe histologic activity (58). In the study of Datz et al. (59), in 2017, one-third of patients with NAFLD were also found with elevated serum ferritin accompanied by normal or mildly increased transferrin saturation.

Recently, in a bioinformatic study, eight ferroptosis-related genes, including ACSL3, ACSL4, AKR1C1, AKR1C2, CS, FADS2, GSS, and PGD were reported elevated in patients with higher hepatic steatosis grade. This evidence indicates the essential role of iron metabolism and ferroptosis plays in NAFLD.

Moreover, there is evidence showing that iron overload correlates with insulin resistance in causing NAFLD. In the year 2006, in insulin-resistant syndrome patients, serum ferritin was found to be correlated with disease severity (60). Insulin resistance is also one of the hallmarks of NAFLD. In year 2008, in vitro, iron chelation deferoxamine was shown to upregulate the phospho-Akt signaling and its downstream Forkhead Box O1 (FoxO1) and Glycogen synthase kinase-3β (Gsk3β) expression and also increase the insulin receptor activity and glucose uptake (61). This further shows that iron overload can result in insulin resistance. The mechanisms of how iron overload affects insulin sensitivity was found in year 2019. Iron long-term overload would suppress Akt-mTOR signaling and further inhibit the regeneration of autophagolysosome resulting in the accumulation of dysfunctional autophagosome, which then in turn impairs the insulin-stimulated uptake and insulin signaling (62). Insulin resistance may elevate the fat accumulation in liver causing NAFLD. Indeed, iron overload was also shown to accelerate the tissue damage and oxidative stress in NAFLD and promote disease progression in high-fat diet mice (63).

For ferroptosis, researchers found that in methionine/choline-deficient diet (MCD) feeding mice, RSL-3 (ferroptosis inducer) can decrease the GPX4 expression and aggravate the disease severity, whereas GPX4 activator sodium selenite and ferroptosis inhibitor liproxstatin-1 (Lip-1) can alleviate the NASH in mice (64). Another independent group found increased lipid reactive oxygen species (ROS) accumulation and ferroptosis in MCD-feeding mice, and the ferroptosis inhibitor ferrostatin-1 (Fer-1), Lip-1, and enoyl coenzyme A hydratase 1 (ECH1) can significantly improve the NASH severity and tissue damage in mice (65, 66). In NASH mouse model induced by choline deficiency and ethionine (CDE) supplementation feed, increased PUFA-PE and ferroptosis would lead to liver damage and inflammatory infiltration. The ferroptosis inhibitor such as antioxidant Trolox can completely reverse the progression of NAFLD (7). Moreover, Tripartite Motif-Containing 59 (TRIM59) was found elevated in NAFLD tissues with potence of promoting disease progression by inducing GPX4 ubiquitination and degradation (67). It indicates the crucial role that ferroptosis plays in NAFLD progression.

However, there is also evidence showing ferroptosis may act as “double-edged sword” in NAFLD progression. Tripartite Motif-Containing 26 (TRIM26) was found to induce ferroptosis by ubiquitination of SLC7A11 to inhibit the System Xc^−^ and GSH synthesis. However, overexpression of TRIM26 can induce ferroptosis in hepatic stellate cell (HSC) and attenuate and improve fibrosis (68), providing a new strategy in the treatment of NAFLD.

## TREATMENT OF NAFLD

So far, there is no regular medicament for NAFLD. Since iron metabolism and ferroptosis was found strongly associated with NAFLD progression, it might be promising to target iron overload and ferroptosis in NAFLD. Some drugs or therapies targeting iron metabolism or ferroptosis are already in clinical trials (Table 1). Antioxidants such as metadoxine, vitamin E and its isoforms are already in phase III/IV now for treating NAFLD. Some drugs treating type 2 diabetes mellitus targeting peroxisome proliferator-activated receptor (PPAR) receptors such as saroglitazar and lobeglitazone was also used in clinical trials for patients with diabetes together with NAFLD. Since PPAR signaling was shown to alleviate ferroptosis in liver (69), these PPAR agonists might also be effective in treating NAFLD. Fenofibrate for treating hypertriglyceridemia might also be promising in the treatment of NAFLD as it can activate Nrf2 signaling.

**Table 1.** The clinical trials of therapies targeting iron metabolism and ferroptosis

Therapies	Target	Function	Phase in Clinical Trail	Trial Identification
Phlebotomy	Iron metabolism	Iron depletion	II	NCT00230087
			II	NCT00641524
			III	NCT01342705
			III	NCT01045525
			III	NCT00658164
Deferasirox	Iron metabolism	Iron chelation	I/II	EUCTR2009-012916-41-DE
			I/II	NCT01278056
Vitamin E	Inhibiting ferroptosis	Antioxidant	III/IV	CTRI/2022/01/039538
			III/IV	CTRI/2022/05/042462
			II	NCT04801849
			II	NCT04198805
			III	NCT04193982
			II	NCT03669133
			II/III	NCT00655018
Tocovid suprabio	Inhibiting ferroptosis	Antioxidant	II	NCT04704063
Tocotrienol	Inhibiting ferroptosis	Antioxidant	II	NCT02581085
α-Tocopherol	Inhibiting ferroptosis	Antioxidant	II	SLCTR/2019/038
δ-Tocopherol	Inhibiting ferroptosis	Antioxidant	II	SLCTR/2019/038
			II	SLCTR/2015/023
Metadoxine	Inhibiting ferroptosis	Antioxidant	IV	NCT02051842
Saroglitazar	Inhibiting ferroptosis	PPARα/γ agonist	III/IV	CTRI/2022/01/039538
			III/IV	CTRI/2022/05/042462
			III	NCT04193982
Lobeglitazone	Inhibiting ferroptosis	PPARγ agonist	IV	NCT02285205
Lanifibranor	Inhibiting ferroptosis	PPARα/γ/δ agonist	II	NCT03459079
Pemafibrate	Inhibiting ferroptosis	PPARα agonist	II	JPRN-jRCTs031200280
Gemcabene	Inhibiting ferroptosis	PPARα agonist	II	NCT03436420
Pemafibrate	Inhibiting ferroptosis	PPARα agonist	II	NCT03350165
Pioglitazone	Inhibiting ferroptosis	PPARγ agonist	II	IRCT20190122042450N5
			III	IRCT20190701044062N3
			III	IRCT2015120819554N10
			III	IRCT201309188308N3
Fenofibrate	Inhibiting ferroptosis	Activation of Nrf2	II	JPRN-jRCTs031200280
			III	IRCT2015120819554N10
Empagliflozin	Inhibiting ferroptosis	Activation of Nrf2	II	IRCT20190122042450N5

The peroxisome proliferator-activated receptor (PPAR) agonists and nuclear factor erythroid 2-related factor 2 (Nrf2) activators are used in patients with type 2 diabetes or hypertriglyceridemia with nonalcoholic fatty liver disease (NAFLD).

### Targeting Iron Metabolism

#### Phlebotomy.

Although favored by barber-surgeon in the medieval, phlebotomy was proved to be ineffective or even harmful in most cases now. In regarding to NAFLD, it was still treated as an effective remedy. A case control study in 2007 first showed that iron depletion by phlebotomy can improve NAFLD damage by improving insulin resistance (70). The subsequent phase II clinical trial also showed a histological improvement in patients with NAFLD after iron depletion by phlebotomy (71). Another study involving 32 patients with NAFLD also showed decreased alanine aminotransferase (ALT), aspartate aminotransferase (AST), and Alkaline Phosphatase (ALP), and improvement in fibrosis, steatosis, and hepatitis after phlebotomy (72).

#### Iron chelator.

Since iron overload can induce insulin resistance which is a main cause of NAFLD, iron chelator such as deferoxamine, deferasirox, and deferiprone can combine with iron to allow its removal from body to reduce the excessive hepatic iron. Deferoxamine has also been proved to alleviate NAFLD in vitro and in vivo (61, 73). In rat, deferasirox was shown to help angiotensin II type I receptor blocker in attenuating NASH (74). In a clinical phase I/II trial (EUCTR2009-012916-41), 2 of 5 patients showed clear improvement. Deferiprone administration even showed a nearly complete improvement in CDE-diet NAFLD mice (7).

### Targeting Ferroptosis

#### Ferroptosis inhibitor.

##### Targeting PUFA peroxidation.

Vitamin E is a lipid-soluble radical-trapping antioxidant that can reduce the oxidation level of membrane PUFAs (75). CoQ10 was also reported to attenuate NAFLD in mouse model (76). Fer-1 and Lip-1 can also inhibit the membrane PUFAs oxidation by inhibiting 15-LOX in mouse model (64–66). Zileuton which is a 5-LOX specific inhibitor can also suppress ferroptosis was shown to reverse the NAFLD progression in rat (77). Meanwhile, rosiglitazone and troglitazone, the PPARγ agonists, can also suppress ACSL4 in PUFA peroxidation in vitro or in vivo in the pilot study (45, 78).

##### Targeting Nrf2.

Since activation of Nrf2 was shown to suppress peroxidation by inducing antioxidant expression. Dimethyl fumarate (DMF), an activator of Nrf2, might also help inhibit NAFLD due to its effect on AFLD (79). Ginkgolide B can also inhibit the ferroptosis by activating Nrf2 signaling to eliminate the free radicals and attenuate NAFLD (80). Dehydroabietic acid (DA), which can bind and activate the upstream Keap1 to promote the expression of Nrf2, might also play a role in scavenging ROS accumulation (81).

#### Ferroptosis inducer.

##### Targeting system Xc.

Erastin and its derivatives piperazine erastin and imidazole ketone erastin can inhibit System Xc^−^ causing GSH depletion. In mouse model, they were found improving liver fibrosis and hepatocellular (82). Meanwhile, sulfasalazine and sorafenib can also cause ROS accumulation in HSCs, which might also be promising in the treatment of liver cancer (83).

##### Targeting other molecules in ferroptosis.

Statins can reduce the activation of CoQ10 to reduce the antioxidant activity and induce the ferroptosis, whereas artesunate can activate ferritinophagy, to induce the release of ferritin bound ferric iron into LIP to induce the ferroptosis (84). Magnesium isoglycyrrhizinate (MgIG) can induce the HO-1 production and promoting the recycling of iron to increase the cytosolic iron and induce the ferroptosis (85).

### Other Promising Targets in Treating NAFLD

In addition to all the aforementioned treatments, there are more ferroptosis regulated genes that are still currently being studied in the laboratory stage. Here, we summarized all published ferroptosis-related genes in liver cells and their primary targeted processes or proteins in ferroptosis so far (Table 2).

**Table 2.** Ferroptosis regulated genes and their key targeted processes or protein reported so far in liver cells

Key Regulator	Reported Genes	PMID
Iron metabolism	*HMOX1*, *FGF21*, *HUWE1*, *TfR1*, *TF*, *ZFP36*, *TAK1*, *STING*, *CAV1*, *NCOA4*, *YAP1*, *ELAVL1*, *TRIB2*, *PCBP1*, *HDAC3*, *PTBP1*, *SIRT1*, *IREB2*, *AGER1*, *IGFBP7*, *IRP1*, *ENO1*, *STEAP3*, *VAMP2*, *EIF3H*, *APOC1*	34530349, 29957462, 37422643, 37707747, 35260822, 32374849, 31679460, 36968217, 37634745, 35489326, 31877357, 34026460, 36398583, 34129254, 36660932, 36182901, 37151632, 30081711, 34315867, 34455040, 32438524, 38034086, 31610175, 32947011, 37280194, 37714372, 35121990, 34790664, 36029649, 37559097, 36108528
Lipid peroxidation	*VDAC1*, *GLS2*, *FADS2*, *FMO1*, *PEX14*, *G6PD*, *ACSL4*, *AMFR*, *PLIN5*, *ECH1*, *GCN5L1*, *POR*, *CYB5R1*, *ALOX12*, *TRPM2*, *OIT3*, *SCD1*, *TXNRD*, *MFN2*	34401974, 35895807, 34520742, 37553313, 37657642, 34325001, 35963845, 38065978, 37349836, 33813878, 37415391, 33321093, 37275121, 36132142, 37805657, 32732975, 36966125
SystemXc-/GSH/GPX4 axis	*SOCS2*, *TGFB1*, *TGFBR1*, *ATF4*, *RRM2*, *PPARA*, *YTHDF1*, *SLC7A11*, *FUNDC1*, *PRDX3*, *FASN*, *BCAT2*, *PNO1*, *TMEM16A*, *ME1*, *BECN1*, *FTO*, *METTL14*, *GPX4*, *TRIM59*, *TRIM26*, *HIC1*, *HNF4A*, *RBM24*, *IDO1*, *BMP4*, *TMSB4X*, *PDK4*, *AKR1C3*, *DAZAP1*, *HIF1A*, *FBXO31*, *IRF1*, *NR1H4*, *HSDL2*, *SRC*, *ENO3*, *AADAC*, *TXNRD3*, *ABCC5*, *USP5*, *SLC12A5*, *PCDHB14*, *C8orf76*, *CTNNB1*, *USP8*, *PSTK*, *METTL9*, *CKB*, *BAP1*, *CST1*, *UCHL3*, *TMEM147*, *DHCR*, *GOT1*	35995846, 32471991, 35202887, 36996941, 35724508, 33372599, 35703725, 34607160, 28195347, 36828120, 37863053, 36604718, 33097833, 36446769, 36572666, 36840630, 35395830, 36232407, 35248719, 37984229, 34609072, 31610178, 36417114, 33869196, 31108460, 36478739, 31945497, 35477568, 34280397, 37582412, 36920042, 33358859, 36878459, 36717099, 36269678, 37441987, 37903763, 37718459, 38077794, 33987359, 36163032, 36934969, 34768109, 37492786, 36645171, 35688944, 35884471, 37311739, 37867237, 34983546, 38017014, 37156912, 30202049, 36369321, 38092274, 37891677, 34381026
FSP1-CoQ10-NAD(P)H axis	*MCT1*, *POR*, *FSP1*	33296645, 32080622, 37741044
Nrf2	*NRF2*, *GSTZ1*, *FDPS*, *SESN2*, *BMAL1*, *FGF4*, *HMGB1*, *CPLX2*, *PCDH20*, *QSOX1*	26403645, 36856923, 36071041, 36482709, 36495698, 33931597, 37100057, 31323261, 36166594, 36702076, 31545489, 36107387, 36641129, 33770521
P53	*BRD7*, *GAS1*, *TRIM23*, *TRIM28*, *URI*	32863216, 37859699, 37495427, 37805657
MAPK/AMPK	*FNDC3B*, *MSI2*, *FARSB*	36336231, 38041016, 38069034
Histone modification	*RB1CC1*, *H3F3A*, *HDAC6*, *HMGCL*, *DPP4*	35220675, 36226351, 37620223, 36508088

However, it should be noted that iron metabolism and ferroptosis are interconnected processes rather than two independent processes. They could affect each other, for example when iron storage increased, there would also be a rise in ferroptosis. Therefore, as the research progresses, the key targeted processes or proteins reported so far might also be altered.

## CONCLUSIONS

**Figure 3.**
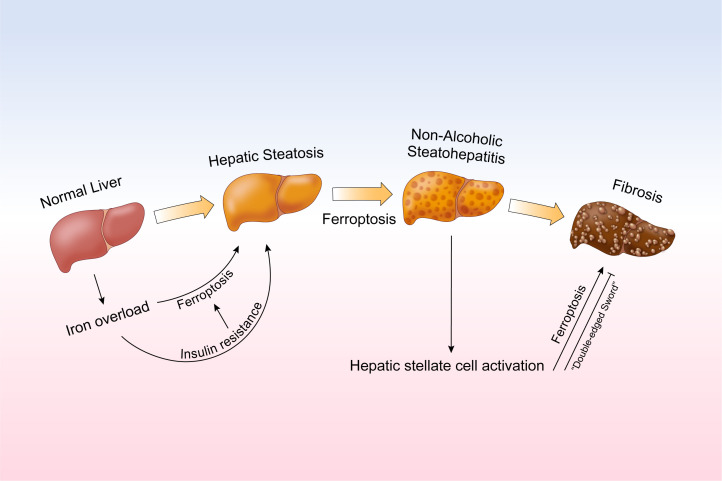
The development of nonalcoholic fatty liver disease (NAFLD) is initiated by iron overload, which results in insulin resistance and ferroptosis and cause the development of severity. Activation of hepatic stellate cell further cause the fibrosis in liver.

Nonalcoholic fatty liver disease is the leading cause of chronic liver disease with increasing cases worldwide. Without intervention, it would develop into a severe form of NASH in the patient. Subsequently, after the HSC activation, fibrosis will be developed. However, there is till now still no FDA approved treatment for NAFLD, although more than seven drugs are now under phase III trial (86). At current stage, weight loss and lifestyle change are still the most effective way in controlling NAFLD. On the other hand, as the research develops, it is thought that the iron overload resulting in insulin resistance and the ferroptosis caused by Fenton reaction would cause the hepatic steatosis and its further progression. However, when developed into fibrosis, ferroptosis may also play a protective role as the ferroptosis in HSC cell may also result in an improvement (Fig. 3). Here, we summarized the clinical and experimental data in regarding to the treatment of NAFLD. The treatment targeting iron metabolism in NAFLD has already been driven to phase II-III trial, whereas the treatment exclusively for NAFLD targeting ferroptosis is still under development as they were still in the mouse model and without any clinical support. Some drugs for diabetes treatment might also play a role as they can regulate ferroptosis by PPAR or Nrf2 signaling. However, the usage of these drugs in patients with NAFLD without diabetes should still be evaluated. Besides, the “double-edged sword” effect of ferroptosis also need to be fully assessed. Even so, iron metabolism, especially ferroptosis should still be considered as the novel key target for the treatment of NAFLD.

## GRANTS

This study is supported by The National Natural Science Foundation of China under Grant No. 81960111 and Natural Science Foundation of Jiangxi Province under Grant No. 20202BABL206013. Z.Y. has been supported by the China Scholarship Council Grant 202008360174, X.S. has been supported by the China Scholarship Council Grant 201909110092.

## DISCLOSURES

No conflicts of interest, financial or otherwise, are declared by the authors.

## AUTHOR CONTRIBUTIONS

X.S. and J.C. conceived and designed research; X.S. and Z.Y. prepared figures; X.S. and Z.Y. drafted manuscript; C.W. and C.H. edited and revised manuscript; X.S., Z.Y., C.W., C.H., and J.C. approved final version of manuscript.
